# Effectiveness and Safety of Switching to Fixed-Dose, Preservative-Free Tafluprost/Timolol in the Treatment of Open-Angle Glaucoma or Ocular Hypertension: A Real-World Study in Taiwan

**DOI:** 10.7759/cureus.87447

**Published:** 2025-07-07

**Authors:** Wei-Wen Su, Yung-Sung Lee

**Affiliations:** 1 Department of Ophthalmology, Chansn Hospital, Taoyuan, TWN; 2 Department of Ophthalmology, Linkou Chang Gung Memorial Hospital, Taoyuan, TWN; 3 Department of Ophthalmology, New Taipei Municipal Tucheng Hospital, New Taipei, TWN; 4 College of Medicine, Chang Gung University, Taoyuan, TWN

**Keywords:** fixed-dose combination, glaucoma, ocular hypertension, preservative-free, tafluprost

## Abstract

Objective

The objective of this study is to evaluate the effectiveness, safety, and tolerability of a preservative-free fixed-dose combination of tafluprost (0.0015%) and timolol (0.5%) (PF tafluprost/timolol FC) in patients with open-angle glaucoma (OAG) or ocular hypertension (OHT).

Methodology

This real-world, prospective, noninterventional study was conducted in Taiwanese patients with OAG or OHT who had an inadequate response or intolerance to topical prostaglandin analogue (PGA) monotherapy and were therefore switched to PF tafluprost/timolol FC. The primary endpoint was the mean change in intraocular pressure (IOP) from baseline to six months after initiating treatment. Changes in clinical signs and subjective symptoms were also evaluated, and adverse events (AEs) were recorded.

Results

A total of 49 patients were enrolled, and 42 completed the study. The mean ± SD IOP at baseline was 16.5 ± 3.5 mmHg, which significantly decreased to 15.4 ± 3.4 mmHg at six months (absolute reduction: 1.1 ± 2.6 mmHg; p < 0.001). The number of patients with a tear break-up time >10 seconds increased significantly from three (7.5%) at baseline to 22 (52.5%) at six months (p < 0.001). However, a nonsignificant increase was observed in subjective symptoms. Six treatment-related AEs were reported, all of which were nonserious and mild to moderate in severity, including contact dermatitis, redness and itchiness, and blurred vision.

Conclusions

This real-world, prospective study in Taiwan demonstrated that switching patients with OAG or OHT from PGA monotherapy to PF tafluprost/timolol FC was effective and safe for reducing IOP.

## Introduction

Glaucoma is a multifactorial disease characterized by progressive optic neuropathies [1,2]. It is the second leading cause of blindness worldwide and the most common cause of irreversible blindness, with an age-standardized global prevalence of 2.29 per 1,000 people [3]. In 2020, the global number of individuals aged over 40 with primary open-angle glaucoma (POAG) was estimated at 68.56 million, with 53.81% residing in Asia [4]. Although data from Taiwan are limited, the prevalence of open-angle glaucoma (OAG) in individuals aged ≥72 years has been reported at 3.7% [5].

Early detection followed by timely treatment can halt or slow the progression of glaucoma to blindness, highlighting the importance of effective care [3]. Elevated intraocular pressure (IOP) is recognized as the most significant, and currently the only modifiable, risk factor for the progression of OAG. Lowering IOP has been shown to reduce both the onset and progression of glaucoma [6].

First-line treatments for reducing IOP typically involve beta-blockers or prostaglandin analogues (PGAs) [6]. However, when monotherapy fails to achieve sufficient IOP reduction, combination therapy is recommended [6]. Fixed-dose combinations (FCs) of a beta-blocker and a PGA are often preferred, as they reduce dosing frequency and enhance patient adherence [7]. Additionally, preservative-free (PF) formulations are favored due to the known toxic effects of preservatives, particularly benzalkonium chloride, which can contribute to ocular surface disease and exacerbate inflammation [8,9].

In Taiwan, the only available PF FC is tafluprost (0.0015%) and timolol (0.5%) (PF tafluprost/timolol FC) [10]. The efficacy and safety of PF tafluprost/timolol FC have been demonstrated in randomized controlled trials, and its real-world performance has been documented in the VISIONARY studies and other European investigations [7,11-15]. However, there is a lack of data regarding its effectiveness, safety, and tolerability in Asian populations.

Therefore, this real-world, prospective, single-center study aimed to evaluate the effectiveness, safety, and tolerability of PF tafluprost/timolol FC in Taiwanese patients with OAG or ocular hypertension (OHT) who were previously treated with topical PGA monotherapy.

## Materials and methods

Study design

This was a real-world, noninterventional, single-center, prospective study conducted at Linkou Chang Gung Memorial Hospital, Taoyuan, Taiwan. The study adhered to the current version of the Declaration of Helsinki and followed the International Conference on Harmonisation Guidelines on Good Clinical Practice. It was registered at ClinicalTrials.gov (NCT04828057; registration date: April 1, 2021) and conducted in accordance with the requirements of the Chang Gung Memorial Hospital Medical Center Institutional Review Board (IRB).

The study protocol and informed consent form were approved by the IRB on December 24, 2020, with a subsequent amendment approved on August 21, 2021. Patient enrollment took place between September 1, 2021, and August 24, 2022.

**Figure 1:**
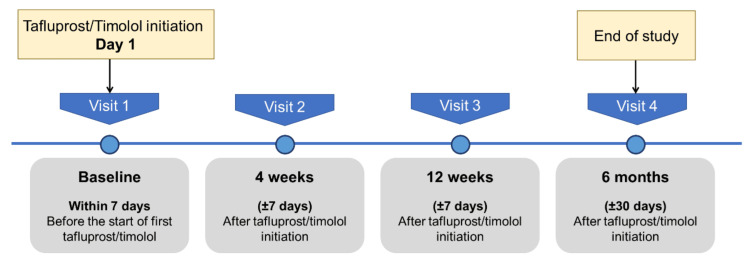
Study visit schedule

The study visit schedule is illustrated in Figure 1. In summary, data were collected prospectively during routine clinical visits at therapy initiation, week 4, week 12, and six months after treatment initiation. All assessments were conducted by the same ophthalmologists (principal investigators). Due to the clinic’s appointment scheduling system, most patients attended follow-up visits at the same time of day as their baseline appointment. Written informed consent was obtained from all participants prior to any study-related screening procedures.

Patients

Patients with OAG or OHT who exhibited an insufficient response to topical PGA monotherapy and were considered suitable for combination therapy were enrolled. Eligibility was determined by the treating ophthalmologists based on their clinical judgment of potential benefit from PF tafluprost/timolol FC. Male and female patients aged ≥20 years who provided written informed consent and had not previously received PF tafluprost/timolol FC were eligible.

Exclusion criteria included pregnancy or breastfeeding, any ophthalmic surgery within six months prior to enrollment, or participation in another investigational study within 30 days prior to enrollment. Patients were also excluded if they had any contraindications listed on the drug label in Taiwan, such as reactive airway disease (including bronchial asthma or a history of asthma), severe chronic obstructive pulmonary disease, sinus bradycardia, sick sinus syndrome (including sinoatrial block), second- or third-degree atrioventricular block not controlled with a pacemaker, overt cardiac failure, or cardiogenic shock [16].

Study treatment

Patients instilled one drop of PF tafluprost/timolol FC into the affected eye(s) once daily, as prescribed by their ophthalmologists [16]. No washout period was implemented for previous medications. The dosing time (morning or evening) was recorded at each study visit.

Study assessments and outcome measures

Following the provision of informed consent, baseline data were collected within seven days prior to treatment initiation. These included demographic information, diagnosis, prior treatment type and duration, and any concomitant nonglaucoma medications, which were also tracked at follow-up visits.

IOP was measured using Goldmann applanation tonometry, either in the morning or afternoon. The study eye was defined as the one with the higher baseline IOP; if both eyes had equal IOP, the right eye was selected for analysis.

This study followed the design of the previously published European VISIONARY study [14]. The primary endpoint was the mean absolute change in study eye IOP from baseline to six months after initiating PF tafluprost/timolol FC. Secondary endpoints included the mean change in IOP from baseline to weeks 4 and 12 and the proportion of responders achieving ≥20% IOP reduction at six months.

Clinical signs were evaluated at each follow-up visit. Conjunctival hyperemia was graded on a four-point severity scale (none, mild, moderate, and severe) [17]. Other assessments included corneal fluorescein staining (CFS; Oxford scale grades 0-V), best corrected visual acuity (BCVA) using the decimal score, Schirmer’s test, and tear break-up time (TBUT; categorized as ≤5 seconds, 6-10 seconds, or >10 seconds). Subjective symptoms - dry eye, irritation, itching, foreign body sensation, and eye pain - were also rated (none, mild, moderate, and severe) at each visit.

Adverse events (AEs) were recorded at every visit. Any concomitant medications potentially associated with AEs were also documented.

Physicians rated effectiveness (IOP control), clinical signs (conjunctival hyperemia, CFS, BCVA, Schirmer’s test, and TBUT), and compliance (based on patient feedback and survey results) using a three-point scale (better, equal, and worse) compared to the previous treatment. Patients assessed treatment tolerability via a questionnaire using a four-point scale (very good, good, satisfactory, and poor).

Statistical analysis

The study aimed to enroll 50 patients. No formal sample size calculation was performed since there were no plans for comparative statistical testing. However, a post hoc power analysis was conducted to confirm the reliability of the findings.

The full analysis set included all patients who were enrolled and received at least one dose of PF tafluprost/timolol FC, regardless of treatment continuation. The effectiveness analysis set included patients from the full analysis set who continued treatment for six months and had at least one IOP measurement at six months (±30 days).

Baseline characteristics and demographics were summarized using mean ± SD or median and IQR for continuous variables (e.g., age, IOP, BCVA, and Schirmer’s test). Categorical variables (e.g., gender, CFS grade, and TBUT category) were summarized using frequencies and percentages. The Shapiro-Wilk test was used to assess normality. Paired t-tests or Wilcoxon signed-rank tests were applied to evaluate changes in primary and secondary outcomes.

McNemar’s test was used to assess changes in categorical subgroups between baseline and follow-up visits. For BCVA analysis, patients with low vision (BCVA <20/70) were excluded. AEs were reported by frequency and percentage. All statistical tests were two-sided, with significance set at p < 0.05. Analyses were performed using SAS version 9.4 (SAS Institute, Cary, NC, USA).

## Results

**Figure 2:**
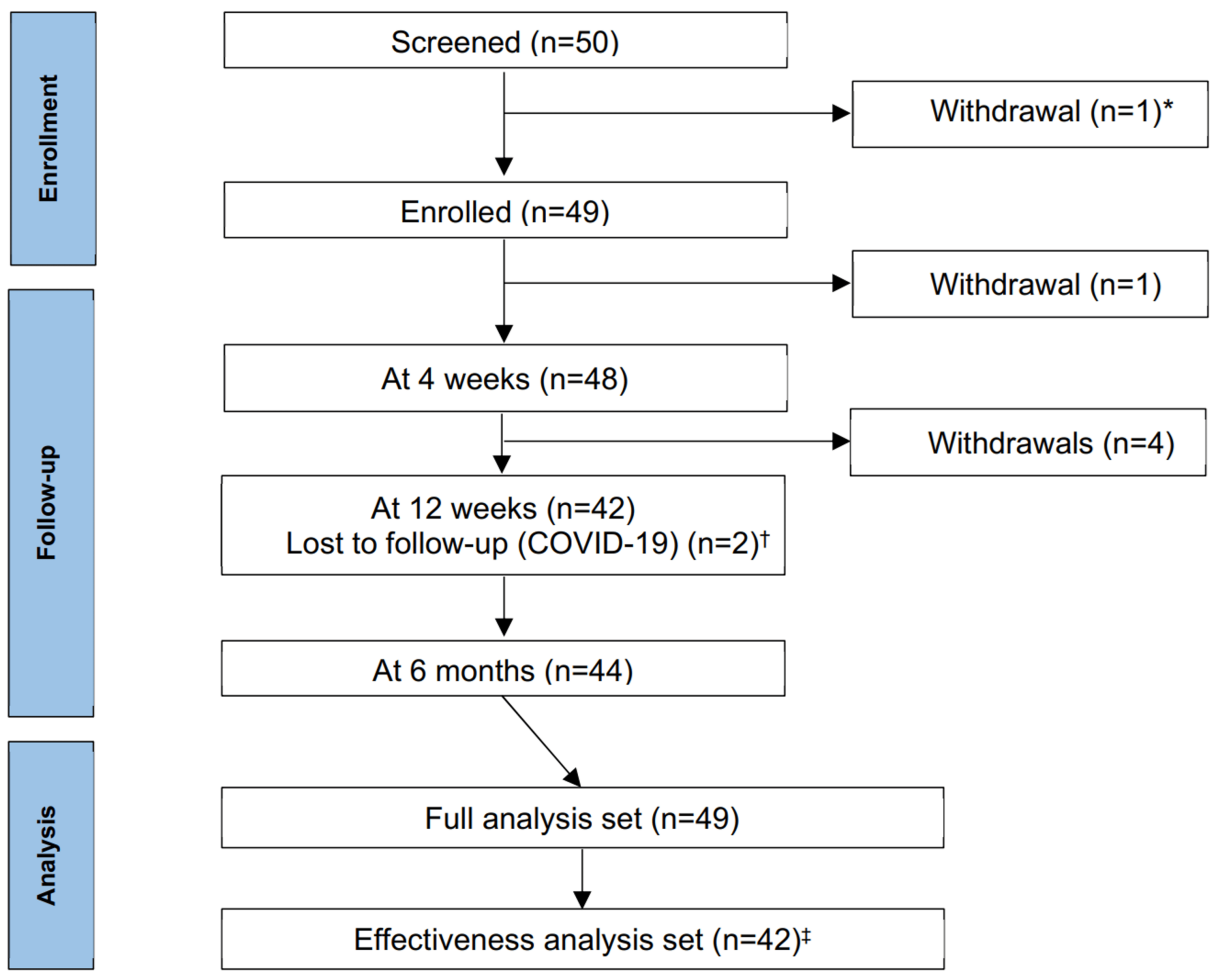
CONSORT flow diagram showing participant flow throughout the study process ^* ^Excluded due to violation of inclusion criteria ^†^ Patients diagnosed with COVID-19 missed the 12-week follow-up visit but did not withdraw from the trial ^‡^ Excluded from the effectiveness analysis set due to discontinuation of treatment before six months and lack of IOP measurement at six months (±30 days) CONSORT: Consolidated Standards of Reporting Trials

The study was conducted from September 1, 2021, to August 24, 2022. One patient who had previously used latanoprost and brimonidine was excluded from follow-up due to a violation of the inclusion criteria (Figure 2). As a result, 49 patients were enrolled, and 42 completed the study. These 49 patients constituted the full analysis set.

Five patients discontinued the study: one each due to blurred vision, conjunctival hyperemia, contact dermatitis, and iritis (the latter not related to treatment), and one due to treatment interruption caused by an accidental suspension of the study drug supply. Additionally, two patients were lost to follow-up after developing COVID-19 infections during the pandemic.

Forty-two patients who continued PF tafluprost/timolol FC treatment for six months and had at least one IOP measurement at six months (±30 days) formed the effectiveness analysis set (Figure 2). A post hoc power analysis based on the mean change in IOP at six months indicated a power of 74.8% with this sample size.

The patients’ mean ± SD age was 59.4 ± 11.1 years (range: 34.7-80.5 years), with 27 (55%) being male (Table 1). The most common diagnosis was POAG in 32 patients (65.3%), followed by OHT and normal tension glaucoma, each in seven patients (14.3%).

**Table 1:** Demographics and baseline characteristics of the study group (N = 49) ^* ^Prostaglandin analogue ^†^ Oxford grade scale BCVA: best corrected visual acuity; CFS: corneal fluorescein staining; IOP: intraocular pressure; OHT: ocular hypertension; POAG: primary open-angle glaucoma; TBUT: tear break-up time

Demographics/characteristics	N = 49
Sex, n (%)	
Male	27 (55.1)
Female	22 (44.9)
Age, years	
Mean ± SD	59.4 ± 11.1
Minimum, maximum	34.7, 80.5
Study eye, n (%)	
Right	26 (53.1)
Left	23 (46.9)
Diagnosis, n (%)	
POAG	32 (65.3)
OHT	7 (14.3)
Normal tension glaucoma	7 (14.3)
Others	3 (6.1)
Previous treatment, n (%)	
Latanoprost^*^	43 (87.8)
Bimatoprost^*^	4 (8.2)
Tafluprost^*^	1 (2.0)
Travoprost^*^	1 (2.0)
Time of IOP measurement, n (%)	
Morning	39 (79.6)
Afternoon	10 (20.4)
IOP (mmHg), mean ± SD	
Study eye	16.5 ± 3.6
Morning	17.0 ± 3.4
Afternoon	14.9 ± 3.8
CFS score^†^, n (%)	
0	22 (44.9)
I	14 (28.6)
II	8 (16.3)
III	4 (8.2)
IV	1 (2.0)
V	0 (0.0)
BCVA (decimal score), mean ± SD	
Study eye	0.6 ± 0.3
Low vision, n (%)	2 (4.1)
Schirmer’s test (mm)	
Median (IQR)	5.0 (6.00)
TBUT seconds, n (%)	
≤5 seconds	22 (44.9)
6-10 seconds	23 (46.9)
>10 seconds	4 (8.2)

All patients had been receiving PGA monotherapy prior to switching to PF tafluprost/timolol FC (Table 1). The most frequently used PGA was latanoprost (n = 43, 87.8%), followed by bimatoprost (n = 4, 8.2%), tafluprost (n = 1, 2.0%), and travoprost (n = 1, 2.0%).

The reasons for switching to PF tafluprost/timolol FC were insufficient IOP control (n = 27, 55.1%), glaucoma progression (n = 9, 18.4%), poor local tolerance of previous treatment (n = 23, 46.9%), poor compliance (n = 3, 6.1%), and dryness (n = 1, 2.0%). Some patients switched for more than one reason.

In the full analysis set, the baseline IOP of the study eye was 16.5 ± 3.6 mmHg (mean ± SD). There was no statistically significant difference between the mean IOP measured in the morning (17.0 ± 3.4 mmHg) and in the afternoon (14.9 ± 3.8 mmHg) (p = 0.09; Table 1).

The most commonly prescribed nonglaucoma medications used in conjunction with glaucoma treatment were artificial tears (19 instances, 32.8%), carbomer gel (11 instances, 19.0%), fluorometholone (nine instances, 15.5%), and lubricating ointment (six instances, 10.3%) (Table 2). By the end of the study, the use of concomitant medications had decreased by 44.8%.

**Table 2:** Summary of concomitant medicines and therapy (N = 49)

Medication/therapy name	Number of times of therapy prescription (%), N = 58
Artificial tears	19 (32.8)
Carbomer gel	11 (19.0)
Fluorometholone	9 (15.5)
Lubricating ointment (Duratears)	5 (8.6)
Emedastine	2 (3.4)
Sulfamethoxazole	2 (3.4)
Prednisolone acetate	2 (3.4)
Betamethasone + neomycin 0.35%	1 (1.7)
Neostigmine methylsulfate	1 (1.7)
Chloramphenicol	1 (1.7)
Ketotifen	1 (1.7)

Change in IOP from baseline

The primary endpoint - change in IOP - was analyzed in the effectiveness analysis set (n = 42). Following the switch to PF tafluprost/timolol FC therapy, the mean ± SD IOP decreased from 16.5 ± 3.5 mmHg at baseline to 15.4 ± 3.4 mmHg at six months, representing a 6.7% reduction (Table 3). This change was statistically significant (p < 0.001), with an absolute IOP reduction of 1.1 ± 2.6 mmHg. At weeks 4 and 12, the absolute IOP reductions were 1.7 ± 2.6 mmHg (10.1%, p < 0.001) and 1.2 ± 3.1 mmHg (7.3%, p = 0.02), respectively (Table 3).

**Table 3:** Changes in IOP from baseline at week 4, week 12, and month 6 (n = 42) ^*^ Significance testing was conducted using a two-sided paired t-test for changes in mean IOP from baseline to each visit. IOP: intraocular pressure

Visit	N	Mean IOP ± SD (mmHg)	Mean IOP reduction from baseline ± SD (mmHg)	Mean IOP reduction from baseline (%)	p-value^*^
Baseline	42	16.5 ± 3.5	-	-	-
Week 4	42	14.8 ± 3.0	1.7 ± 2.6	10.1	<0.001
Week 12	42	15.3 ± 3.4	1.2 ± 3.1	7.3	0.02
Month 6	42	15.4 ± 3.4	1.1 ± 2.6	6.7	<0.001

Among patients who were switched from latanoprost monotherapy (n = 35), the mean IOP significantly decreased by 1.04 ± 2.6 mmHg (6.4%, p = 0.02) from baseline to six months. At weeks 4 and 12, the mean IOP reductions in this subgroup were 1.56 ± 2.8 mmHg (9.6%, p = 0.002) and 1.21 ± 3.3 mmHg (7.4%, p = 0.04), respectively.

**Figure 3:**
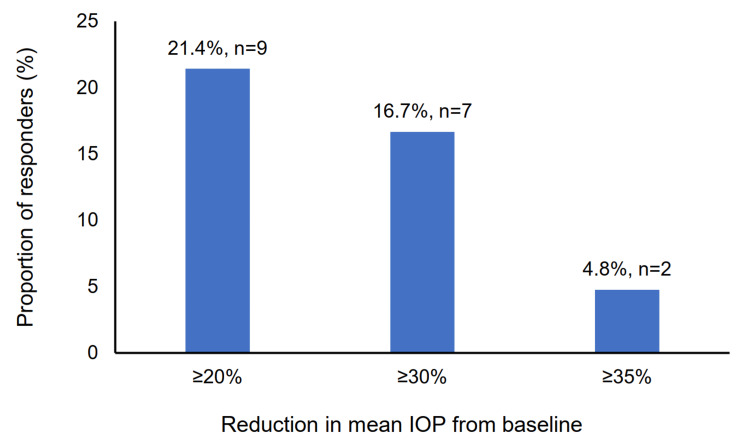
Proportion of patients with ≥20% reduction in mean IOP at six months following therapy initiation IOP: intraocular pressure

At six months, the responder rate, defined as the proportion of patients achieving a ≥20% reduction in mean IOP from baseline, was 21.4% (Figure 3).

Changes in clinical signs and symptoms

**Figure 4:**
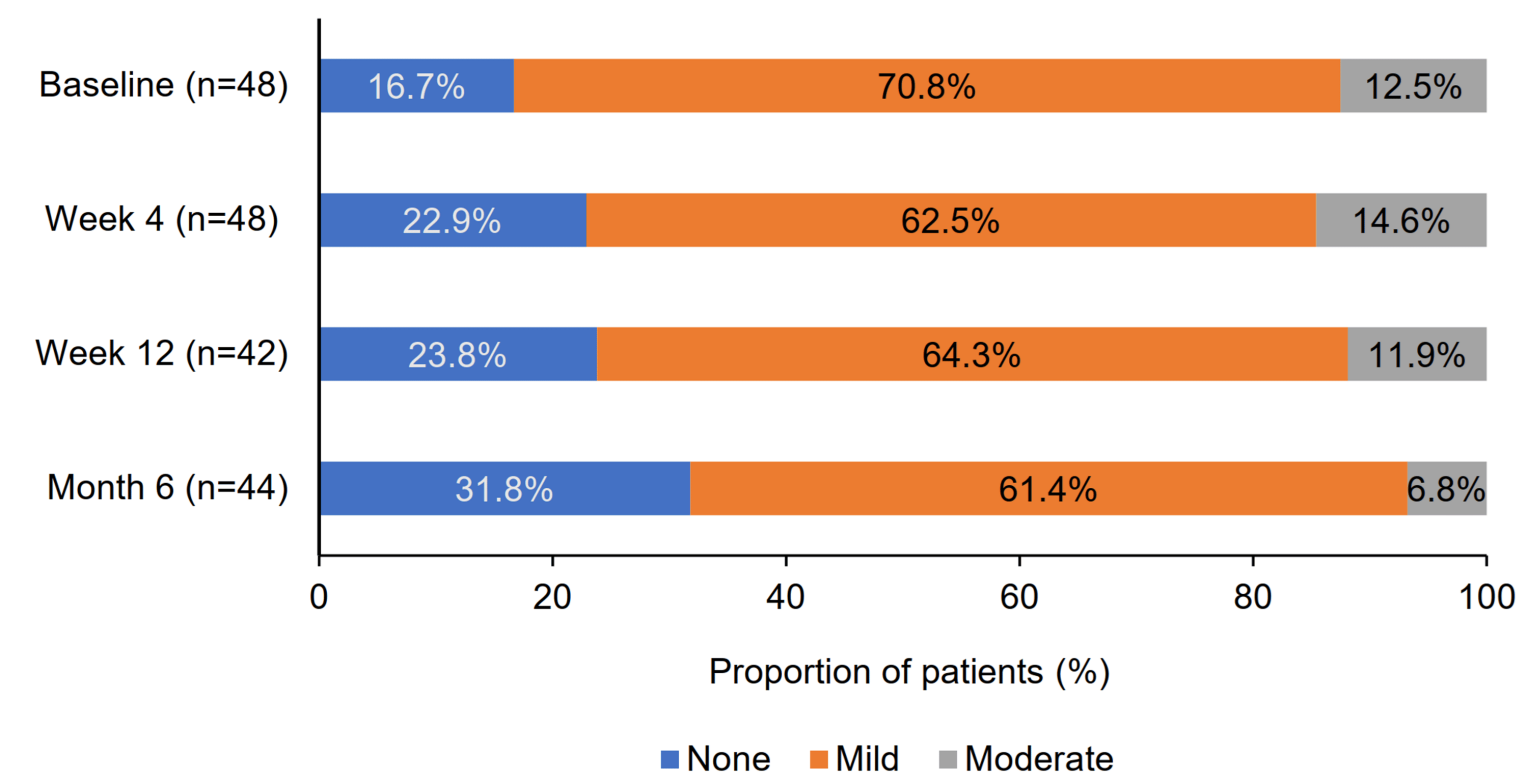
Changes in proportions of patients with conjunctival hyperemia severity

Changes in clinical signs and symptoms were evaluated in both the full analysis set (n = 49) and the effectiveness analysis set (n = 42). In the full analysis set, the number (proportion) of patients without conjunctival hyperemia increased from eight (16.7%) at baseline to 14 (31.8%) at month 6. A reduction was also observed in patients with mild hyperemia, from 34 (70.8%) to 27 (61.4%), and with moderate hyperemia, from six (12.5%) to three (6.8%) (Figure 4).

In the effectiveness analysis set, moderate or severe conjunctival hyperemia was observed in six (14.3%) patients at baseline, decreasing to five (11.9%) at weeks 4 and 12, and to three (7.1%) at month 6. These reductions were not statistically significant.

**Figure 5:**
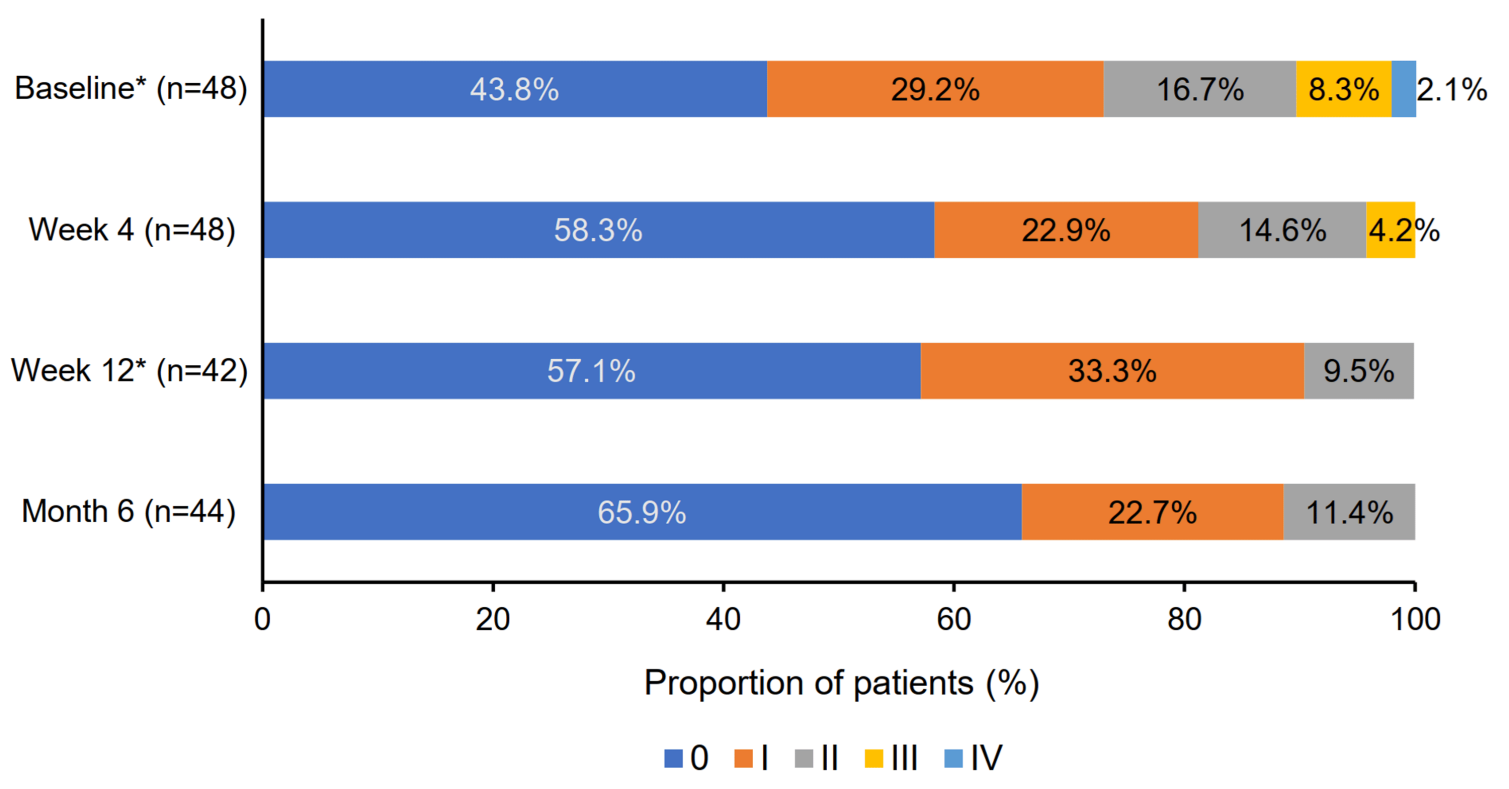
Changes in the proportion of patients with CFS scores according to the Oxford grading scale ^*^ At baseline and week 12, patient proportions may not total 100% due to rounding. CFS: corneal fluorescein staining

In the full analysis set, patients with a CFS Oxford grade score of 0 increased from 21 (43.8%) at baseline to 29 (65.9%) at month 6. Notably, no patients had a CFS score of grade III or IV by month 6 (Figure 5). 

Among patients in the effectiveness analysis set, five (11.9%) had a CFS grade of III/IV/V at baseline. This number dropped to two (4.8%) at week 4 and to 0 (0.0%) at both week 12 and month 6, although the reductions were not statistically significant.

A significant mean decrease of 0.1 ± 0.2 in the BCVA decimal score was observed from baseline (0.64 ± 0.3) to month 6 (0.57 ± 0.3) (p = 0.03). No significant changes were observed in Schirmer’s test results (baseline: 6.4 ± 4.9 mm; month 6: 6.1 ± 4.2 mm; p = 0.73) at any follow-up visit.

**Figure 6:**
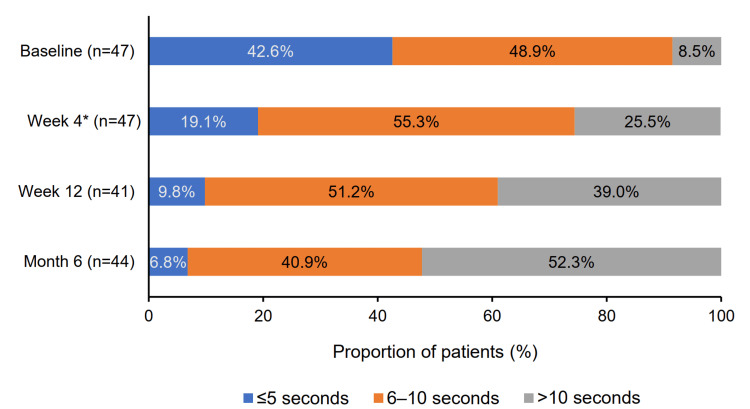
Changes in proportion of patients across TBUT categories ^*^ At week 4, patient proportions may not total 100% due to rounding.

In the effectiveness analysis set, the number (proportion) of patients with TBUT >10 seconds significantly increased from three (7.5%) at baseline to 11 (27.5%) at week 4 (p = 0.01), 15 (37.5%) at week 12 (p < 0.001), and 22 (52.5%) at month 6 (p < 0.001). In the full analysis set, patients with TBUT ≤5 seconds and 6-10 seconds decreased from 20 (42.6%) and 23 (48.9%) at baseline to three (6.8%) and 18 (40.9%) at month 6, respectively (Figure 6).

Changes in subjective symptoms

**Figure 7:**
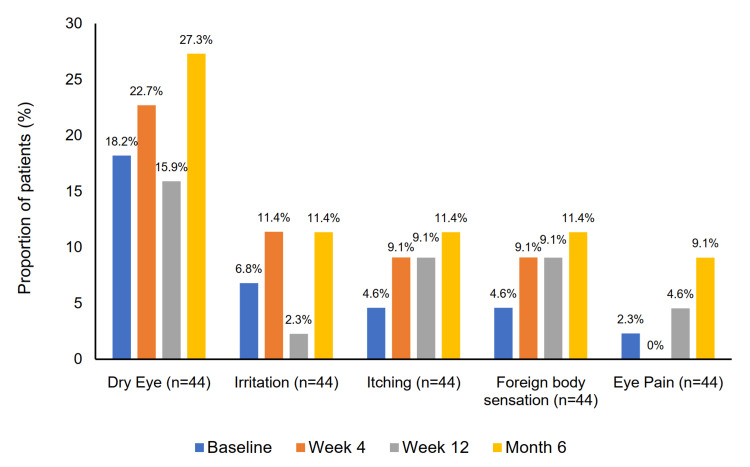
Changes in the proportion of patients reporting moderate to severe subjective symptoms

The proportion of patients experiencing moderate to severe subjective symptoms - including dry eye, irritation, itching, foreign body sensation, and eye pain - increased over the study period, but none of these changes reached statistical significance at any visit (Figure 7). Moderate to severe dry eye was reported in eight patients (18.2%) at baseline and in 12 patients (27.3%) at six months (p = 0.25). Moderate to severe irritation increased from three patients (6.8%) at baseline to five patients (11.4%) at six months (p = 0.48). Itching was reported as moderate/severe in two patients (4.6%) at baseline and in five patients (11.4%) at six months (p = 0.26). Moderate to severe foreign body sensation was reported in two patients (4.6%) at baseline and five patients (11.4%) at six months (p = 0.18). Eye pain of moderate to severe intensity was reported by one patient (2.3%) at baseline, increasing to four patients (9.1%) at six months (p = 0.08).

Physicians’ and patients’ assessments

Physicians evaluated the effectiveness of PF tafluprost/timolol FC therapy compared with previous treatments based on IOP control, changes in clinical signs, and patient compliance. At six months, the therapy was rated as more effective in 15 patients (30.6%) and equally effective in 24 patients (49.0%). Clinical signs were assessed to have improved in 26 patients (53.1%) and remained unchanged in 18 patients (36.7%). Better compliance was reported in 21 patients (42.9%) and similar compliance in 22 patients (44.9%) compared with prior therapy. Additionally, 40 patients (81.6%) rated the tolerability of PF tafluprost/timolol FC as satisfactory, good, or very good.

Safety outcomes and discontinuation from study treatment

A total of six treatment-related AEs were reported in five patients during the six-month study period (Table 4). All AEs were nonserious and ranged from mild to moderate in severity. Three patients discontinued the study due to AEs. One patient developed moderate contact dermatitis, which resolved with treatment. Another experienced moderate redness and itchiness, which were ongoing at the time of discontinuation. The third patient reported moderate blurred vision, which resolved with treatment. No discontinuations were due to insufficient IOP control.

**Table 4:** Treatment-related AEs reported during the study period (N = 49) AE: adverse event

System organ class	Number of events	Intensity
Eye disorders		
Foreign body sensation	1	Mild
Vision blurred	1	Moderate
Eye pruritus (allergic reaction)	1	Mild
Conjunctival hyperemia	1	Moderate
Skin and subcutaneous tissue disorders		
Pruritus (allergic reaction)	1	Moderate
Contact dermatitis	1	Moderate

## Discussion

To the best of our knowledge, this is the first real-world, prospective study in Taiwan to evaluate the effectiveness and safety of PF tafluprost/timolol FC therapy after switching from PGA monotherapy in patients with OAG or OHT.

At six months post-switch, the mean IOP significantly decreased by 6.7% (1.1 ± 2.6 mmHg), and 21.4% of patients achieved an IOP reduction of ≥20%. The proportion of patients with TBUT >10 seconds also significantly increased, though no other clinical signs or subjective symptoms showed statistically significant changes. Physicians assessed PF tafluprost/timolol FC as safer than the previous treatment, and over 80% of patients reported being satisfied with the therapy after switching.

The IOP reduction observed in this study (6.7%) was lower than that reported in VISIONARY studies conducted in Europe (23.6%) [14], Spain (23.5%) [11], and Italy (22.7%) [13], all involving patients previously on PGA monotherapy. Similarly, the responder rate (≥20% IOP reduction) in our study was lower compared to Europe (69.2%) [14], Spain (62.0%) [11], and Italy (68.1%) [13]. A possible explanation for this difference is the lower mean baseline IOP in our study population (16.5 ± 3.5 mmHg) compared to those in the VISIONARY studies in Europe (21.4 ± 4.5 mmHg) [14], Italy (19.5 ± 3.7 mmHg) [13], and Spain (22.2 ± 3.8 mmHg) [11].

According to baseline monotherapy treatment in the VISIONARY study, IOP reductions from baseline to month 6 were 5.4 ± 4.0 mmHg in PGA users and 6.6 ± 4.2 mmHg in beta-blocker users [12]. A systematic review by Holló et al. demonstrated a linear relationship between baseline IOP and the extent of IOP reduction with fixed-dose tafluprost/timolol, although the studies analyzed included a washout period [18]. In our study, patients switched to PF tafluprost/timolol FC due to insufficient IOP control (55.1%), poor tolerance (46.9%), glaucoma progression (18.4%), poor compliance (6.1%), or dry eye symptoms (2.0%) from the previous medication. These factors may explain the relatively lower IOP reduction and responder rate in our cohort. Nevertheless, it is promising that additional IOP reduction was achieved despite the already low baseline IOP after PGA monotherapy.

The significant improvement in TBUT could be attributed to the PF formulation. In contrast, the absence of improvement in subjective dry eye symptoms might be due to the pharmacological differences between PGAs and the addition of timolol. Previous studies have reported a weak correlation between subjective and objective dry eye measures [19]. A significant decrease in BCVA decimal score was observed at six months (from 0.64 ± 0.3 to 0.57 ± 0.3; p = 0.03). While BCVA remained stable in the Spain and Italy VISIONARY studies, a nonsignificant increase was seen in the UK study [7,11,13]. Instillation time varied across regions: in Spain, Italy, and the UK, most patients administered the drops in the evening (97%, 85%, and 67%, respectively) [11,13,20], whereas all patients in our study instilled the drops in the morning. This likely reflects regional clinical practices and physician recommendations. Given that common side effects of PGAs include eyelid edema, blurred vision, and dry eyes [21,22], it is plausible that the observed decline in visual acuity was related to patients undergoing follow-up assessments shortly after their morning instillation.

A 44.8% reduction in the use of concomitant medications was observed by study end, suggesting a decreased need for additional therapies to manage adverse effects typically associated with PGAs, such as dry eye.

The incidence of treatment-related AEs in our study (13.6%) was lower than that reported in other VISIONARY studies (Europe: 53.5%; Spain: 50.0%) [11,14]. Although these AEs were not linked to treatment failure, their influence on patient-reported outcomes and adherence cannot be fully excluded. The ocular AEs observed in this study, such as contact dermatitis and blurred vision, have been previously reported in both clinical trials and real-world settings [9]. Contact dermatitis, often an allergic reaction to timolol, may be misdiagnosed if not carefully evaluated, though symptoms generally improve upon discontinuation of the drug [23,24]. Blurred vision has been reported in 0.1% to <5% of patients treated with PF tafluprost/timolol FC [9,18,25,26]. Clinical data suggest most AEs are ocular, mild to moderate, and nonserious [16].

More than 70% of patients in this study were already using lubricants (e.g., artificial tears, carbomer gel, and Duratears) for dry eyes, suggesting preexisting ocular surface issues prior to switching therapy. Daily exposure to active ingredients and preservatives is known to exacerbate ocular surface disease in glaucoma patients [27]. Notably, the significant TBUT improvement observed after switching supports the potential benefit of PF tafluprost/timolol FC on dry eye signs. At study end, most patients (81.6%) rated the tolerability of the new treatment as satisfactory, good, or very good.

Preservatives such as benzalkonium chloride are known to have cytotoxic effects and can trigger inflammation, reducing the quality of life and adherence to glaucoma treatment [9]. Additionally, dry eye incidence has been positively correlated with the number of glaucoma medications used [28]. The observed improvements in IOP and TBUT, along with favorable physician and patient assessments, suggest that PF tafluprost/timolol FC could enhance both treatment adherence and quality of life in clinical practice.

Unlike randomized controlled trials, we did not include a washout period before switching therapy to better reflect real-world clinical conditions [25,29]. Thus, the outcomes of this study offer valuable insights into the real-world response and tolerability of PF tafluprost/timolol FC in Taiwanese patients.

Limitations

This study had some limitations. Despite observed improvements in IOP and clinical signs, its single-arm design without a control group prevents us from isolating the effects of the PF formulation versus the beta-blocker component. Furthermore, since latanoprost is the most frequently prescribed PGA in Taiwan, our cohort predominantly included patients previously treated with this drug. The small sample size limited subgroup analyses on patients switched from other PGAs (bimatoprost, travoprost, and tafluprost). In addition, Taiwan’s national health guidelines recommend initiating PGAs only when beta-blockers are ineffective or not tolerated [28], meaning our study exclusively evaluated patients previously on PGA monotherapy. This differs from European VISIONARY studies, where patients switched from either beta-blockers or PGAs [14]. Although the efficacy and real-world effectiveness of PF tafluprost/timolol FC have been previously documented in Europe [14], this is the first prospective study in Asia to evaluate its effectiveness, safety, and tolerability after a switch from PGA monotherapy without a washout period.

## Conclusions

This first real-world, prospective study in Taiwan demonstrates that switching from PGA monotherapy to PF tafluprost/timolol FC is effective and safe for patients with OAG or OHT. Physicians rated the treatment as safer than prior therapies, and the majority of patients reported high levels of satisfaction.
